# Correlation of monocyte counts with clinical outcomes in idiopathic nonspecific interstitial pneumonia

**DOI:** 10.1038/s41598-023-28638-5

**Published:** 2023-02-16

**Authors:** Tae Hun Kim, Hyung-Jun Kim, Myung Jin Song, Byoung Soo Kwon, Yeon Wook Kim, Sung Yoon Lim, Yeon Joo Lee, Young-Jae Cho, Jae Ho Lee, Jin-Haeng Chung, Jong Sun Park

**Affiliations:** 1 Divisions of Pulmonary and Critical Care Medicine, Department of Internal Medicine, Seoul National University College of Medicine, Seoul National University Bundang Hospital, 82 Gumi-Ro 173 Beon-Gil, Bundang-Gu, Seongnam, Gyeonggi-Do 13620 South Korea; 2 Department of Pathology, Seoul National University College of Medicine, Seoul National University Bundang Hospital, Seongnam, South Korea

**Keywords:** Diseases, Medical research, Risk factors

## Abstract

Higher blood monocyte counts are related to worse survival in idiopathic pulmonary fibrosis. However, studies evaluating the association between blood monocyte counts and clinical outcomes of idiopathic nonspecific interstitial pneumonia (iNSIP) are lacking. We evaluated the impact of monocyte counts on iNSIP prognosis. iNSIP patients (n = 126; median age, 60 years; female, n = 64 [50.8%]) diagnosed by surgical lung biopsy were enrolled and categorized into low (monocyte < 600/µL) and high (monocyte ≥ 600/µL) monocyte groups. The median follow-up duration was 53.0 months. After adjusting for age, sex, and smoking history, the annual decline in forced vital capacity (FVC) showed differences between the monocyte groups (*P*_interaction_ = 0.006) (low vs. high; − 28.49 mL/year vs. − 65.76 mL/year). The high-monocyte group showed a worse survival rate (*P* = 0.01) compared to low monocyte group. The 5-year survival rates were 83% and 72% in the low- and high-monocyte groups, respectively. In the Cox-proportional hazard analysis, older age, male sex, low baseline FVC, and diffusing capacity of the lung for carbon monoxide were independent risk factors for mortality. However, monocyte count (Hazard ratio 1.61, *P* = 0.126) was not an independent prognostic factor. Although high monocyte count might be associated with faster lung function decline, it could not independently predict survival in iNSIP.

## Introduction

Interstitial lung disease (ILD) is a group of respiratory diseases characterized by varying degrees of inflammation and fibrosis of the lung parenchyma^1,2^. Idiopathic pulmonary fibrosis (IPF) is a progressive fibrotic ILD characterized by decreased lung function and short life expectancy^3^. In addition to IPF, some other ILDs, such as nonspecific interstitial pneumonia (NSIP), unclassifiable idiopathic interstitial pneumonia, connective tissue disease-related ILD (CTD-ILD), and hypersensitivity pneumonitis, also have progressive fibrotic features that are similar to those of IPF^4^.

NSIP is a type of ILD that usually affects female nonsmokers aged 40–60 years. NSIP can be idiopathic or is associated with secondary conditions, such as CTDs, environmental or occupational exposures, drugs, or viral infections. If there is no secondary cause, NSIP is classified as idiopathic NSIP (iNSIP)^5^. Although studies on the natural course of iNSIP are scarce, some studies have shown that the prognosis of NSIP is favorable when compared with that of IPF^6^. Corticosteroids and immunosuppressive agents are widely used and are thought to be beneficial in patients with NSIP^6,7^. However, some patients experience progressive fibrosis despite immunosuppressive treatment^8,9^.

Recent studies have focused on the role of innate immunity in the initiation and propagation of the fibrotic cascade in the lungs and have reported that higher blood monocyte counts may be associated with prognosis or lung function decline and worse survival in adults with IPF^10^. Notably, monocyte-derived macrophages have emerged as an important driver of lung fibrogenesis^11^. Histologically, fibrotic NSIP is characterized by architectural distortion caused by confluence or marked expansion of fibrotic alveolar walls, which tends to be associated with fibroblast foci, but is not accompanied by honeycombing unlike IPF^12^. Therefore, we hypothesized that monocyte count can predict the prognosis of iNSIP. However, no research has investigated the relationship between the prognosis of iNSIP and monocyte counts. This study aimed to evaluate the association between blood monocyte counts and clinical outcomes of iNSIP.

## Results

### Baseline characteristics of patients

We identified and analyzed the data from 126 patients with iNSIP (Table 1). The median follow-up duration was 53.0 (29.0–84.0) months. The mean age and smoking history did not differ between the low- and high-monocyte groups. However, there were more male patients in the high-monocyte group than in the low-monocyte group (*P* = 0.027). There was no statistically significant difference in comorbidity, baseline bronchoalveolar lavage (BAL) lymphocytosis (*P* = 0.350), or baseline pulmonary function test (PFT) results (forced vital capacity [FVC; % of predicted], *P* = 0.104) and diffusing capacity of the lung for carbon monoxide (DL_CO_) [% of predictive], *P* = 0.074) between the low- and high-monocyte groups.

**Table 1:** Characteristics of patients according to blood monocyte counts.

	All patients (N = 126)		*P* value
	Low monocyte (monocyte counts < 600/µL, n = 73)	High monocyte (monocyte counts ≥ 600/µL, n = 53)	
Age, years	59.7 (± 11.2)	60.5 (± 11.0)	0.716
Sex, female	43 (58.9%)	21 (39.6%)	0.027
Smoking history			0.377
Ever smoker Never smoker	26 (35.6%) 47 (64.4%)	23 (43.4%) 30 (56.6%)	
Comorbidity			
Diabetes Hypertension	14 (19.2%) 12 (16.4%)	18 (34.0%) 12 (22.6%)	0.061 0.385
Bronchoalveolar lavage			
WBC, cells/μL Neutrophil, % Lymphocyte, % Macrophage, %	195.0 (105.0–350.0) 6.0 (2.0–17.0) 14.0 (5.0–27.5) 68.5 (50.2–79.8)	150.0 (50.0–280.0) 9.0 (4.0–21.3) 8.5 (3.5–23.0) 70.0 (50.0–86.5)	0.071 0.170 0.350 0.592
Pulmonary Function Test			
FVC, L FVC, % predicted DL_CO_, mL/min/mmHg DL_CO_, % predicted	2.54 (± 0.79) 78.7 (± 17.7) 13.0 (± 3.5) 69.8 (± 15.6)	2.55 (± 0.88) 73.4 (± 17.7) 12.4 (± 4.9) 63.9 (± 21.1)	0.974 0.104 0.467 0.074
Laboratory findings			
WBC, cells/μL	7079 (± 1998)	8828 (± 1883)	< 0.001
Monocyte, cells/ μL	442.0 (± 107.6)	752.0 (± 121.4)	< 0.001
CRP, mg/dl	1.0 (± 1.8)	1.3 (± 1.9)	0.149
Histologic findings			
Fibrotic	31 (42.5%)	26 (49.1%)	0.763
Cellular	39 (53.4%)	25 (47.2%)	
Intermediate	3 (4.1%)	2 (3.8%)	
Medical treatments			
Steroids, n	66 (90.4%)	47 (88.7%)	0.752
Immunosuppressants*, n	13 (17.8%)	19 (35.5%)	0.063
Clinical course			
Relapse**, n	19 (26.0%)	21 (39.6%)	0.106
Acute exacerbation, n	5 (6.8%)	16 (30.2%)	0.001

Data are expressed as the mean (± standard deviation), median (interquartile range), or counts (%).

FVC, forced vital capacity; DL_CO_, diffusing capacity of the lung for carbon monoxide; WBC, white blood cells; CRP, C-reactive protein. NSIP, nonspecific interstitial pneumonia.

*Medications including azathioprine, mycophenolate mofetil, or tacrolimus.

**Deterioration of clinical course including lung function, symptoms, or radiologic findings and re-prescribed steroids with or without immunosuppressants.

Histologic findings revealed fibrotic NSIP pattern in 45.2% (57 out of 126) of the patients, and the proportion of fibrotic NSIP was not different between the low- and high-monocyte groups. Moreover, there was no statistical difference in the medical treatments of steroids with or without other immunosuppressants, including azathioprine, mycophenolate mofetil, or tacrolimus, and relapse rate between the low- and high-monocyte groups (Table 1). In addition, there were significant differences in age (*P* = 0.034), baseline PFT including FVC (% of predicted) (*P* = 0.001) and DL_CO_ (% of predicted) (*P* = 0.001), immunosuppressant therapy (*P* = 0.032), relapse rate (*P* = 0.016), and the proportion of patients with acute exacerbation (< 0.001) between survivors and non-survivors (Table 2).

**Table 2:** Comparison of characteristics between the survivors and non-survivors among the patients with idiopathic nonspecific interstitial pneumonia.

Demographic characteristics	All patients (N = 126)		*P* value
	Survivors (n = 93)	Non-survivors (n = 33)	
Age, years	58.8 (± 11.1)	63.6 (± 10.3)	0.034
Female sex	49 (52.7%)	15 (45.5%)	0.475
Smoking history			0.945
Ever smoker Never smoker	36 (38.7%) 57 (61.3%)	13 (39.4%) 20 (60.6%)	
Comorbidity			
Diabetes Hypertension	23 (24.7%) 14 (15.1%)	9 (27.3%) 10 (30.4%)	0.773 0.055
Bronchoalveolar lavage			
WBC, cells/μL Neutrophil, % Lymphocyte, % Macrophage, %	180.0 (90.0–300.0) 8.0 (3.0–17.0) 11.0 (4.0–26.0) 69.0 (51.0–83.0)	140.0 (52.5–217.5) 7.0 (2.0–21.0) 10.0 (5.0–23.0) 70.0 (50.0–87.0)	0.203 0.845 0.899 0.969
Baseline pulmonary function test			
FVC, L FVC, % predicted DL_CO_, mL/min/mmHg DL_CO_, % predicted	2.68 (± 0.86) 79.5 (± 18.3) 13.6 (± 4.2) 70.5 (± 18.4)	2.17 (± 0.56) 67.9 (± 13.1) 10.4 (± 2.9) 58.4 (± 15.2)	0.002 0.001 < 0.001 0.001
Laboratory findings			
WBC, cells/μL	7525 (± 2010)	8633 (± 2266)	0.010
Monocyte counts, cells/µL	559.0 (± 188.0)	612.9 (± 194.7)	0.164
CRP, mg/dl	1.0 (± 1.7)	1.6 (± 2.2)	0.097
Medical treatments			
Steroids, n	81 (87.1%)	32 (97.0%)	0.109
Immunosuppressants*, n	19 (20.4%)	13 (39.4%)	0.032
Clinical course			
Relapse**, n	24 (25.8%)	16 (48.5%)	0.016
Acute exacerbation, n	9 (9.7%)	12 (36.4%)	< 0.001

Data are expressed as the mean (± standard deviation), median (interquartile range), or counts (%).

FVC, forced vital capacity; DL_CO_, diffusing capacity of the lung for carbon monoxide; WBC, white blood cells; CRP, C-reactive protein.

*Medication including azathioprine, mycophenolate mofetil, or tacrolimus.

**Deterioration of clinical course including lung function, symptoms, or radiologic findings and re-prescribed steroids with or without immunosuppressants.

### Lung function changes according to monocyte count

**Figure 1:**
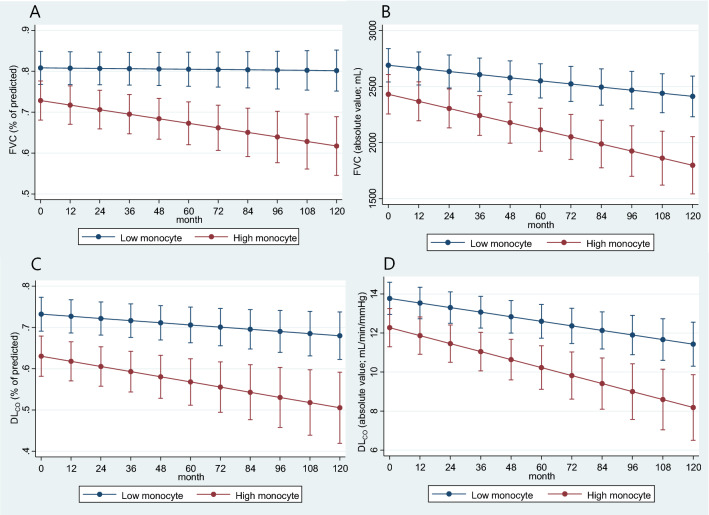
Changes in mean pulmonary function over time with a linear mixed-effects model in the study patients according to monocyte count group. (**A)** Change of FVC (% of predicted). (**B)** Change of FVC (mL). (**C)** Change of DL_CO_ (% of predicted). (**D)** Change of DL_CO_ (mL/min/mmHg). Each bar represents 95% confidence interval.

The average lung function change over 10 years was calculated using the regression analysis of the linear mixed-effect model after adjusting for age, sex, and smoking history, and it was found that the annual decline of predicted FVC (mL) was -28.49 mL/year and − 65.76 mL/year in the low- and high-monocyte groups, respectively (Table 3). FVC (mL) (*P* = 0.006) and FVC (% of predicted) (*P* = 0.005) showed a significant interaction over time between the low- and high-monocyte groups (Fig. 1A, B). The annual decline of predicted DL_CO_ (mL/min/mmHg) was − 0.26 mL/min/mmHg/year and − 0.40 mL/min/mmHg/year in the low- and high-monocyte groups, respectively. The predicted DL_CO_ (% of predicted) was − 0.73%/year and − 1.16%/year in the low- and high-monocyte groups, respectively (Fig. 1C, D). However, there was no significant interaction over time for DL_CO_ (mL/min/mmHg) (*P* = 0.068) or DL_CO_ (% of predicted) (*P* = 0.144) (Table 3).

**Table 3:** Annual average changes in lung function in the included patients according to blood monocyte counts.

	Low monocyte (monocyte counts < 600/µL)		High monocyte (monocyte counts ≥ 600/µL)		*P*_interaction_
	Annual decline	95% CI	Annual decline	95% CI	
FVC (mL)	− 28.49 mL/year	− 41.48 to − 15.51	− 65.76 mL/year	− 87.57 to − 43.95	0.006
FVC (% of predicted)	− 0.06%/year	− 0.44 to 0.30	− 1.11%/year	− 1.74 to − 0.48	0.005
DL_CO_ (mL/min/mmHg)	− 0.26 mL/min/mmHg/year	− 0.33 to − 0.19	− 0.40 mL/min/mmHg/year	− 0.51 to − 0.28	0.068
DL_CO_ (% of predicted)	− 0.73%/year	− 1.09 to − 0.37	− 1.16%/year	− 1.75 to − 0.56	0.144

CI, confidence interval; FVC, forced vital capacity; DL_CO_, diffusing capacity of the lung for carbon monoxide.

The interaction according to time between high and low monocyte groups was analyzed using the type III test, and the *P* value of the test was expressed as *P*_interaction_.

### Clinical course and survival according to monocyte count

**Figure 2:**
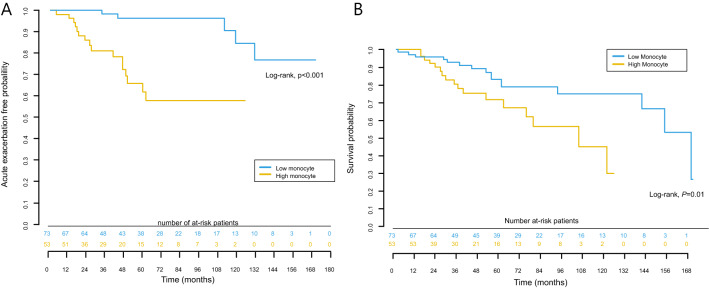
(**A)** Kaplan-Meier curves of time to first acute exacerbation for the patients with idiopathic nonspecific interstitial pneumonia according to monocyte counts. (**B)** Kaplan–Meier survival curves of the patients with idiopathic nonspecific interstitial pneumonia according to monocyte counts.

The proportion of patients who experienced relapse tended to be high in the high-monocyte group, but there was no statistical difference between the two groups (low vs. high: 26.0% vs. 39.6%; *P* = 0.106). The proportion of patients with a history of acute exacerbation was higher in the high-monocyte group than in the low-monocyte group (Table 1). Time to first event of acute exacerbation in the low- and high-monocyte groups is shown in Fig. 2A. The high-monocyte group had a shorter time to first event of acute exacerbation than the low-monocyte group (*P* < 0.001).

Patients with higher monocyte counts had significantly lower survival rates than those with lower monocyte counts (log-rank test, *P* = 0.01) (Fig. 2B). The median survival times were 168 and 104 months in the low- and high-monocyte groups, respectively. The 5-year survival rates were 83% and 72% in the low- and high-monocyte groups, respectively. For patients who died in the hospital, death was mainly due to respiratory causes associated with acute exacerbation (Supplementary Table S1).

Cox proportional hazard model was performed to find predictors of mortality, including age, sex, smoking history, baseline BAL lymphocytes, baseline lung function (i.e., FVC [% of predicted] and DL_CO_ [% of predicted]), and monocyte group (Table 4). A high monocyte count (hazard ratio [HR] = 2.46, 95% confidence interval [CI]: 1.19–5.10, *P* = 0.016) was a significant predictor of mortality based on the univariable analysis. However, in the multivariable analysis, a high monocyte count (HR = 1.61, 95% CI: 0.88–2.97, *P* = 0.126) was not a significant predictor of mortality after adjusting for age, sex, and baseline pulmonary function (Table 4). Older age (HR = 1.08, *P* < 0.001), male sex (HR = 2.23, *P* = 0.038), low baseline FVC (% predicted) (HR = 0.98, *P* = 0.037), and DL_CO_ (% predicted) (HR = 0.97, *P* = 0.002) were significant predictors of mortality in iNSIP (Table 4). High monocyte count was not an independent predictor of mortality when the Cox proportional hazard model was applied separately to the fibrotic (n = 57) and cellular NSIP (n = 64) subgroups (Supplementary Tables S2 and S3).

**Table 4:** Cox proportional hazard analysis of mortality in patients with idiopathic nonspecific interstitial pneumonia.

Characteristics	Univariable			Multivariable		
	HR	95% CI	*P* value	HR	95% CI	*P* value
Age	1.05	1.19–5.10	0.016	1.08	1.05–1.11	< 0.001
Male sex	2.25	1.09–4.68	0.029	2.23	1.04–4.75	0.038
Ever smoking	1.58	0.76–3.28	0.218	–	–	**–**
BAL lymphocyte, %	1.00	0.98–1.03	0.938	–	–	–
High monocyte	2.46	1.19–5.10	0.016	1.61	0.88–2.97	0.126
FVC (% of predicted)	0.96	0.94–0.99	0.001	0.98	0.96–0.99	0.037
DL_CO_ (% of predicted)	0.97	0.95–0.99	0.003	0.97	0.95–0.99	0.002

HR, hazard ratio; CI, confidence interval; BAL, bronchoalveolar lavage fluid; FVC, forced vital capacity; DL_CO_, diffusing capacity of the lung.

## Discussion

In this study, we evaluated the long-term clinical course of a homogeneous group of iNSIP patients and analyzed the impact of blood monocyte count on the prediction of the clinical course of iNSIP. The mechanistic rationale for our study is as follows: monocytes can migrate to damaged tissues to aid tissue repair^13^. However, this process could become detrimental and result in fibrotic changes when tissue damage is continuous. Increased CD14 + classical monocytes in the peripheral blood correlate with poor outcomes in patients with IPF^14^, and they differentiate into profibrotic macrophages, which contribute to pulmonary fibrosis. Some patients with iNSIP progress to “progressive pulmonary fibrosis” despite treatment and have a similar clinical course as IPF^15^.Therefore, this study evaluated the effect of monocyte count on the prognosis of iNSIP.

We found that a higher monocyte count at baseline was related with a faster decline in FVC. However, a higher monocyte count had no relationship with a faster decline in DL_CO_ and was not an independent prognostic factor for mortality. In a previous study of fibrosing lung disease, the adjusted rate of decline in FVC was − 187.8 mL/year^16^; in another study of fibrotic NSIP, lung function was improved or stable in 80% of the patients, and reduced FVC at 12 months was a predictor of mortality^6^. However, in our study, iNSIP patients showed a persistent decrease in mean FVC and DL_CO_, although the decrease was not as much as that in fibrosing lung disease.

Time-to-event analysis showed higher rates of acute exacerbation and mortality in the high-monocyte group than in the low-monocyte group among the iNSIP patients. However, a high monocyte count was not an independent predictor of mortality after adjusting for age, sex, and baseline PFT in Cox proportional hazard model.

The potential reasons why a high monocyte count was not identified as an independent prognostic factor, even if it was a predictor of the rapid decline of FVC, are as follows. A rapid decrease in FVC (≥ 10%) or DLCO (≥ 15%) is associated with the prognosis of ILD^17,18^. In our study, although the decrease in FVC was rapid in the high-monocyte group, the annual decline rate of FVC according to the monocyte group was minimal and did not show as much difference as the criteria for progressive fibrosing ILD mentioned above (low vs. high: − 28.49 mL/year vs. − 65.76 mL/year and − 0.06%/year vs. − 1.11%/year). Therefore, a minimal decrease in FVC would not lead to consequent worse survival. Second, mortality might have been caused by acute exacerbation or other causes rather than by gradual decline of lung function. The proportion of patients with acute exacerbation was higher among non-survivors than among survivors and the high-monocyte group had more patients with a history of acute exacerbation compared to the low-monocyte group. Third, unlike IPF, lymphocyte-related inflammatory response could be more important than monocyte-derived fibrotic process in the pathophysiology of iNSIP. A previous genetic study reported that NSIP cases showed significant enrichment of genes related to the mechanisms of immune reaction, such as T-cell response and recruitment of leukocytes into the lung compartment. In contrast, the enriched genes in IPF were involved in senescence, epithelial-to-mesenchymal transition, myofibroblast differentiation, and collagen deposition^19^. We found that monocyte count was not a predictor of mortality in the subgroup analyis of patients with fibrotic NSIP (n = 57).

In the survival analysis, the high-monocyte group showed better survival than the low-monocyte group within the first 24 months. There were three cases of early mortality in the low-monocyte group; the first case was due to a sudden cardiac arrest from a non-respiratory cause. The other two deaths occurred out of our hospital, and thus we could not identify the cause of death. However, the baseline FVC of one case was 51%; thus, the mortality might be related to poor baseline lung function instead of the monocyte count.

According to a previous study, the 10-year and 5-year survival rates of iNSIP were 73% and 82%, respectively^5^; despite the favorable survival outcomes, some patients experienced deterioration of symptoms and progressive fibrosing diseases^20^. Another study also showed that patients with NSIP could develop acute exacerbation, and the estimated 1-year incidence of acute exacerbation of NSIP was 4.2%^21^. A previous study reported that relapse of NSIP occurred in 36% (20 of 55) of fibrotic NSIP patients who were stable following treatments^20^ and other study on pathologically confirmed NSIP including CTD-ILD reported a relapse rate of 25%^8^. In our study, 40 (31.7%) patients experienced relapse during the follow-up period and required the administration of steroids with or without additional immunosuppressants again. The median survival in our study was 153 months, which was better than that of previous studies, which reported a median survival of 53 months for fibrotic NSIP^6,22^. Notably, a recent study showed that 5-year and 10-year survival was 94.6% and 90.5%, respectively in fibrotic NSIP^23^, which are better than those in our study (75.8% and 61.0%, respectively); this inconsistency might be because that study included 31% of patients with CTD-ILD, and the median age of the included patients was 54 years, which was younger than that in our study^23^.

A previous study evaluated the prognostic factors for fibrotic NSIP and found that preserved PFT, lymphocytosis of BAL fluid, and treatment with steroids and azathioprine might be associated with a lower risk of disease progression^23^. Another study showed that treatment with corticosteroids with or without immunosuppressants might improve lung function, including FVC and DL_CO_, in iNSIP^7^. A recent study reported that decreased baseline DL_CO_ < 60% of predicted was a risk factor for poor prognosis^23^. This finding is similar to that in our study, which also showed that lower baseline DL_CO_ was a significant risk factor for mortality.

This study has several limitations. First, this was a retrospective single-center study; thus, there is a possibility that confounding factors might have influenced the outcomes of our study. Second, this study had a limited sample size and lacked radiological data. More extensive prospective studies are needed to evaluate the clinical value of blood monocytes in predicting outcomes in patients with non-IPF ILD, including iNSIP. Despite these limitations, our study is the first to investigate whether monocyte count is a prognostic factor for iNSIP and provides valuable information about the long-term changes in pulmonary function and the clinical course of patients with iNSIP.

## Conclusions

This study indicated that age, sex, and baseline PFT, including FVC and DL_CO_, were statistically related to survival, and a high monocyte count was a risk factor for the decline in FVC but do not have an independent relationship with survival in patients with iNSIP. The findings suggest that peripheral blood monocyte count may be a useful biomarker to predict the decline in FVC in the routine clinical practice in patients with iNSIP. Further prospective studies are needed to clarify whether blood monocytes are a prognostic factor for iNSIP.

## Methods

### Study design and population

**Figure 3:**
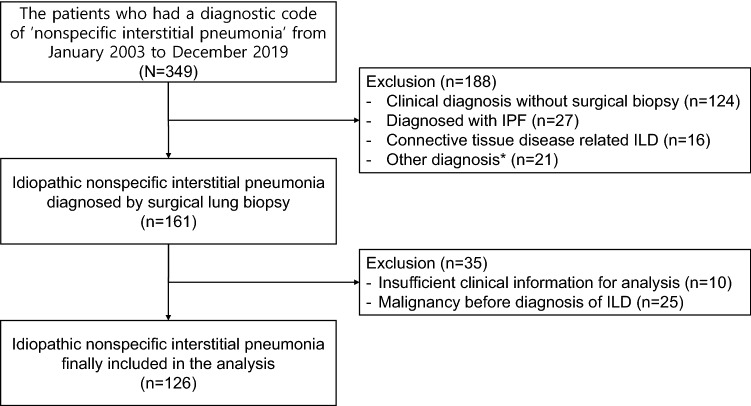
Flow diagram of the study population. *Other diagnoses included cryptogenic organizing pneumonia, chronic hypersensitivity pneumonitis, pleuroparenchymal fibroelastosis, and unclassifiable ILD.

We identified 349 consecutive patients with a diagnostic code of ‘nonspecific interstitial pneumonia’ in the electronic medical records of a tertiary teaching hospital in South Korea from January 2003 to December 2019. We excluded 124 patients without surgical lung biopsy and 64 patients with other diagnoses, including IPF and CTD-ILD. A total of 161 patients had surgical biopsy-proven iNSIP, which was diagnosed based on a multidisciplinary discussion by pulmonologists, radiologists, pathologists, and rheumatologists in some cases. After excluding 35 patients with insufficient clinical information or a history of malignancy, 126 patients with iNSIP were finally included in the analysis (Fig. 3).

### Objectives and outcome variables

This study aimed to determine the relationship between monocyte count at diagnosis and clinical outcomes in patients with iNSIP. Mortality data were obtained from the Ministry of the Interior and Safety. Patients who underwent lung transplantation were regarded as non-survivors based on the transplant date. The basic medical history; laboratory test results including complete blood cell counts, C-reactive protein (CRP), and differential cell counts of BAL fluid; baseline PFT; medical treatment; and clinical course were collected from electronic medical records. Peripheral blood monocyte counts and other laboratory test results were collected at the time of diagnosis before treatment initiation. BAL was performed at the time of initial diagnosis, and PFT was performed every 3–6 months during follow-up. Patients were categorized based on the monocyte count into low (monocyte < 600/µL) and high (monocyte ≥ 600/µL) monocyte groups, as described previously^24^. We only used a monocyte cut-off count of 600/µL due to the small sample size. The distribution of monocyte counts is shown in Supplementary Figure S1. We compared the changes in lung function and survival between the low- and high-monocyte groups. “Relapse” was defined by deterioration of clinical course, including lung function, symptoms, or radiologic findings, and re-prescribed steroids with or without immunosuppressants. “Acute exacerbation” was defined as acute respiratory deterioration with new, bilateral ground glass opacities or consolidation that could not be fully explained by cardiac failure or fluid overload^25^.

### Statistical analysis

All values are presented as mean ± standard deviation (SD), median (interquartile range), or number (%). We used the Kaplan–Meier method to calculate cumulative survival rates and univariate and multivariable Cox proportional hazard models to analyze predictors of mortality. We used the log-rank test to compare the survival rates between the patient groups. Variables with a *p*-value of < 0.2 in the univariate analysis were entered into the multivariable analysis and selected by the backward log-likelihood ratio method. For secondary outcomes, the trends in PFT over time were analyzed using a linear mixed-effects model during the follow-up periods after adjusting for age, sex, and smoking history. The interaction according to time between low- and high- monocyte groups was analyzed using the type III test, and *the P*-value of the test was expressed as *P*_interaction_. Annual lung function changes were calculated using regression analysis of the linear mixed-effects model, assuming an interaction by time. For all analyses, we set a *P*-value of < 0.05 as the limit of statistical significance. All analyses were performed using SPSS version 25.0 (SPSS Inc., Chicago, IL, USA) and Stata version 17 (STATA Corp, College Station, TX, USA).

### Ethics approval and consent to participate

The study protocol was approved by the Institutional Review Board (IRB) of Seoul National University Bundang Hospital (IRB No. B-2205-755-103). The study was conducted in compliance with the principles of the Declaration of Helsinki. The requirement for informed consent was waived by the IRB of the Seoul National University of Bundang Hospital because of the retrospective nature of the study.

## Supplementary Information

Supplementary Information 1.

## Data Availability

The data that support the findings of this study are not openly available because consent to share data was not obtained from the participants. However, the datasets used and analyzed in the current study are available from the corresponding author upon reasonable request.
