# Supplementary material for: Correlation of monocyte counts with clinical outcomes in idiopathic nonspecific interstitial pneumonia
Source: Sci Rep. 2023 Feb 16;13:2804. doi: 10.1038/s41598-023-28638-5 (PMC9935501; doi:10.1038/s41598-023-28638-5)
Supplement: Supplementary file 1 — Supplementary Information 1. [file 41598_2023_28638_MOESM1_ESM.docx]

**Supplementary data**

**Supplementary Figure S.1** Distribution of participants according to monocyte counts


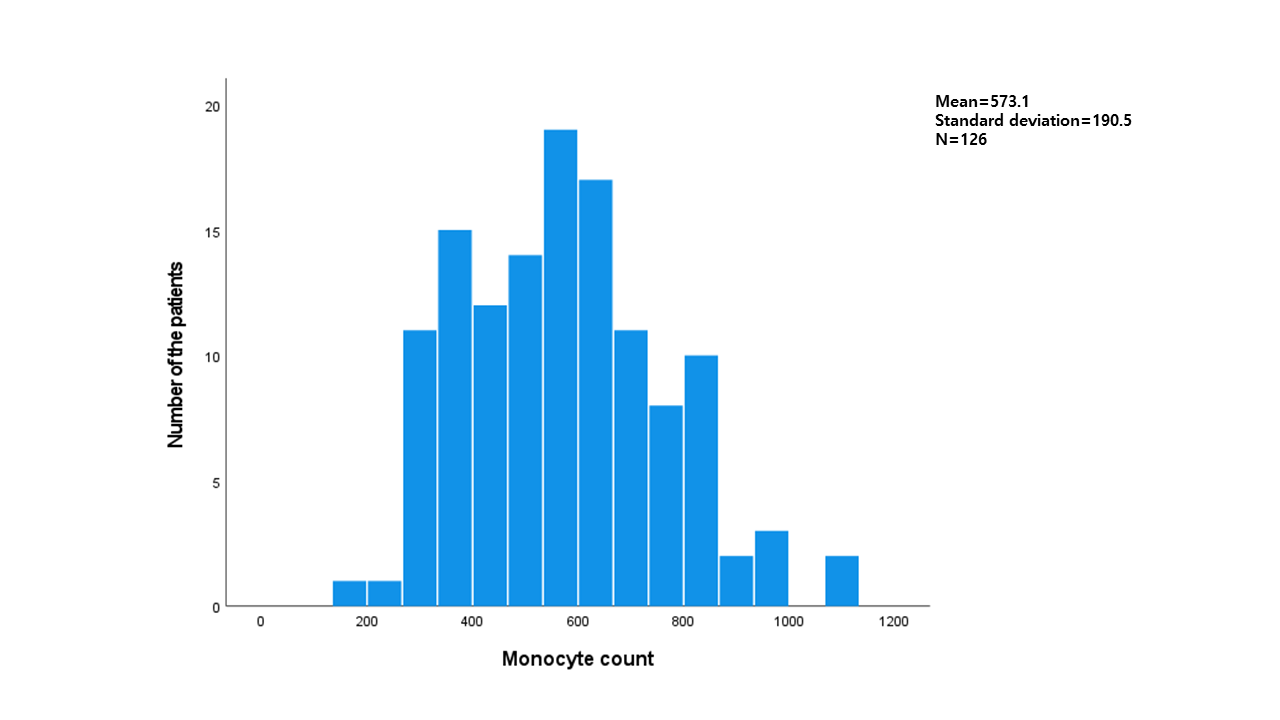


**Supplementary Table S.1** Cause of death among study participants

|  | n = 33 |
| --- | --- |
| Out of hospital mortality | 19 (57.6%) |
| In-hospital mortality | 14 (42.4%) |
| Respiratory  Sepsis  Cardiac arrest  Death on arrival | 10 (33.3%)  2 (6.0%)  1 (3.0%)  1 (3.0%) |

Data are expressed as counts (%)

**Supplementary Table S.2** Cox proportional hazard analysis of mortality in patients with fibrotic iNSIP (n=57)

| Characteristics | Univariable | | |  | Multivariable | | |
| --- | --- | --- | --- | --- | --- | --- | --- |
|  | HR | 95% CI | *P*-value |  | HR | 95% CI | *P*-value |
| Age | 1.05 | 0.99–1.10 | 0.055 |  | 1.05 | 1.00–1.10 | 0.070 |
| Male | 2.46 | 0.98–6.17 | 0.056 |  | 1.57 | 0.60–4.12 | 0.357 |
| High monocyte | 2.74 | 1.10–6.83 | 0.029 |  | 1.65 | 0.61–4.50 | 0.328 |
| FVC (% of predicted) | 0.95 | 0.93–0.98 | 0.002 |  | 0.97 | 0.94–1.00 | 0.055 |
| DL_CO_ (% of predicted) | 0.97 | 0.94–0.99 | 0.017 |  | 0.98 | 0.95–1.01 | 0.212 |

HR, hazard ratio; CI, confidence interval; FVC, forced vital capacity; DL_CO_, diffusing capacity of the lung for carbon monoxide; iNSIP, idiopathic nonspecific interstitial pneumonia.

**Supplementary Table S.3** Cox proportional hazard analysis of mortality in patients with cellular NSIP (n=64)

| Characteristics | Univariable | | |  | Multivariable | | |
| --- | --- | --- | --- | --- | --- | --- | --- |
|  | HR | 95% CI | *P*-value |  | HR | 95% CI | *P*-value |
| Age | 1.03 | 0.98–1.09 | 0.277 |  | 1.03 | 0.97–1.10 | 0.349 |
| Male | 1.70 | 0.43–6.67 | 0.445 |  | 3.86 | 0.71–20.80 | 0.116 |
| High monocyte | 2.31 | 0.62–8.65 | 0.212 |  | 2.04 | 0.46–9.01 | 0.347 |
| FVC (% of predicted) | 0.97 | 0.93–1.02 | 0.198 |  | 0.99 | 0.94–1.04 | 0.618 |
| DL_CO_ (% of predicted) | 0.96 | 0.92–1.00 | 0.069 |  | 0.96 | 0.91–1.01 | 0.138 |

HR, hazard ratio; CI, confidence interval; FVC, forced vital capacity; DL_CO_, diffusing capacity of the lung for carbon monoxide; NSIP, nonspecific interstitial pneumonia.
